# The Profile of the Obstetric Patients with SARS-CoV-2 Infection According to Country of Origin of the Publication: A Systematic Review of the Literature

**DOI:** 10.3390/jcm10020360

**Published:** 2021-01-19

**Authors:** Yolanda Cuñarro-López, Pilar Pintado-Recarte, Ignacio Cueto-Hernández, Concepción Hernández-Martín, María Pilar Payá-Martínez, María del Mar Muñóz-Chápuli, Óscar Cano-Valderrama, Coral Bravo, Julia Bujan, Melchor Álvarez-Mon, Miguel A. Ortega, Juan Antonio De León-Luis

**Affiliations:** 1 Department of Public and Maternal and Child Health, School of Medicine, Complutense University of Madrid, 28040 Madrid, Spain; yolanda.cunarro@gmail.com (Y.C.-L.); ppintadorec@yahoo.es (P.P.-R.); ignaciocuetohernandez@gmail.com (I.C.-H.); chermartin@telefonica.net (C.H.-M.); mpilar.paya@salud.madrid.org (M.P.P.-M.); mar.mchg@gmail.com (M.d.M.M.-C.); jaleon@ucm.es (J.A.D.L.-L.); 2 Department of Obstetrics and Gynecology, University Hospital Gregorio Marañón, Madrid 28009, Spain; 3 Health Research Institute Gregorio Marañón, 28009 Madrid, Spain; 4 Faculty of Medicine, Complutense University of Madrid, 28009 Madrid, Spain; oscarcanovalderrama@hotmail.com; 5 Department of Surgery, Medical and Social Sciences, Faculty of Medicine and Health Sciences, University of Alcalá, 28801 Madrid, Spain; cbravoarribas@gmail.com; 6 Service of Gynecology and Obstetrics, Central University Hospital of Defense-UAH, Madrid 28047, Spain; 7 Unit of Histology and Pathology, Department of Medicine and Medical Specialities, Faculty of Medicine and Health Sciences, University of Alcalá, 28801 Alcalá de Henares, Spain; mjulia.bujan@uah.es (J.B.); mademons@gmail.com (M.Á.-M.); 8 Ramón y Cajal Institute of Sanitary Research (IRYCIS), 28034 Madrid, Spain; 9 University Center for the Defense of Madrid (CUD-ACD), 28047 Madrid, Spain; 10 Immune System Diseases-Rheumatology, Oncology Service an Internal Medicine, University Hospital Príncipe de Asturias, (CIBEREHD), 28806 Madrid, Spain; 11 Cancer Registry and Pathology Department, Hospital Universitario Principe de Asturias, 28806 Madrid, Spain

**Keywords:** COVID-19, gestation, systematic review, maternal-perinatal outcomes, maternal-perinatal morbidity and mortality, maternal characteristics and COVID-19, country profile

## Abstract

SARS-CoV-2 is the novel member of coronavirus responsible for the worldwide pandemic COVID-19, affecting all types of people. In this context, established research identified pregnant women as a susceptible group of SARS-CoV-2 infection, although there is still limited data regarding the real impact of COVID-19 in this group. With that purpose, we conducted a systematic review describing the maternal-fetal results of pregnant women infected by SARS-CoV-2, in aim to analyze the profile of the obstetric patients according to the country of origin of the publication. A total of 38 articles were included in this systematic review with 2670 patients from 7 countries, with 20 works published from China (52.6%). We reported significative differences according to the median maternal age, with Spain as the country with the highest age (34.6 years); The percentage of tabaquism; proportion of symptomatic patients in the triage; type of radiological exam (China and France conduct CT scans on all their patients in comparison to the use of chest X-Ray in the rest of the countries studied); percentages of C-sections (83.9% in China; 35.9% Spain, *p* < 0.001); maternal mortality rate, proportion of patients who need treatments, the use of antivirals, antibiotics, and anticoagulants as well as measurements of the newborns. Perinatal results are favorable in the majority of countries, with very low rates of vertical transmission in the majority of works. The studies collected in this review showed moderate to high index of quality. The different works describe the affectation during the first wave of the pandemic, where the pregnant woman with SARS-CoV-2 infection is generally symptomatic during the third trimester of gestation along with other factors associated with worse prognosis of the disease, such as higher age, body mass index, and further comorbidities developed during pregnancy. In the obstetric patient, proportion of C-sections are elevated together with prematurity, increasing maternal perinatal morbimortality. Differences found between countries could be based on the proper profile of the patient in each region, the period of the pandemic directly affecting how it was managed, and the variations regarding in situ medical attention.

## 1. Introduction

COVID-19 is the accepted term used for the infection performed by the novel coronavirus SARS-CoV-2 suddenly discovered in Wuhan, China [1]. Nowadays, COVID-19 constitutes a global threat considered as an unprecedent pandemic since March 2020. As of 15 December 2020, a total of 71,351,695 cases of COVID-19 have been registered and 1,612,372 associated deaths worldwide, with the Americas (both North and South America) the hardest-hit continent (30,656,971 confirmed cases), followed by Europe (22,116,845 confirmed cases) and South-East Asia (11,430,955 confirmed cases) [2]. SARS-CoV-2 infects human cells through its binding via spike protein to the angiotensin-converting enzyme 2 [ACE-2] receptor [3]. This receptor is widely expressed in various tissues and organs and sex, age, race, or pathological conditions may regulate the expression of ACE-2 [4]. Fever, dry cough, fatigue, and other respiratory difficulties are the most common clinical manifestations, with altered levels of C reactive protein [CRP], lymphocytes, and lactate dehydrogenase [5]. According to its severity, COVID-19 may be stratified from asymptomatic to mild and moderate presentations [in 80% of people], severe [15%], and critical, approximately in 5% of cases [6]. Despite elder people [7] and patients with baseline comorbidities or receiving different therapies [8] being the most vulnerable population, this condition affects all range of ages, including children and obstetric patients. 

In this great context, pregnant women seem to be a particularly susceptible group that may be affected by COVID-19, which may bring unfavorable consequences for both mother and fetus, like preterm birth, preeclampsia, C-section deliveries, and perinatal death [9]. In fact, it is known that in normal pregnancy, ACE-2 is overexpressed, along with an altered immune status, that favors SARS-CoV-2 infection [10,11], therefore showing the urgent need to continue unravelling this association. An increasing amount of studies have compared the impact of COVID-19 in different countries, finding substantial differences in the incidence, prevalence, and fatality of COVID-19, according to multiple variables like the encompassed area, the total and population density, median age, GDP per capita, and the urbanization of the territory [12,13,14]. In this line, a recent review conducted by Figueiro-Filho E. et al. [15] collected the effect of 10,966 pregnant women with COVID-19 in 15 different countries regarding maternal characteristics, clinical symptoms, maternal and neonatal outcomes and they suggested that pregnant women are not more affected by the respiratory complications of COVID-19, when compared to the outcomes described in the general population. However, this earlier review does not study the relation between maternal country and the pregnant women profile presenting SARS-CoV-2 infection. With that purpose, we conducted a systematic review of the literature about the maternal perinatal results in pregnant women infected by SARS-CoV-2, in an aim to describe and analyze the profile of the obstetric patients according to the country of origin of the publication.

## 2. Material and Methods

This systematic review was performed according to the Preferred Reporting Items for Systematic Reviews and Meta-Analyses (PRISMA) statement [16] and Meta-analyses Of Observational Studies in Epidemiology (MOOSE) guidelines for meta-analyses and SR of observational studies [17]. This study was registered in the International Prospective Register of Systematic Reviews (PROSPERO) database [registration number: CRD 42020219959]. 

The systematic review search was conducted in 2 electronic databases, PubMed/MEDLINE and Web of Science on 28 September 2020 using combinations of the relevant medical subjects with the following keywords: “SARS-CoV-2” or “COVID-19” AND “pregnancy” AND “humans”. A reference database (EndNoteX9, Thomson-Reuters) was used to incorporate all references. 

Inclusion criteria were original articles, case reports, case series, and randomized controlled trials that described women of any age affected by SARS-CoV-2 during pregnancy or the postnatal period. The included articles had to report about the country of origin of the publication and they had to inform at least 10 cases of obstetric patients with SARS-CoV-2 infection. There were no publication date or language restrictions on the search and references of relevant papers were searched manually for relevant studies. 

Studies were excluded if they did not report data of number of cases of SARS-CoV-2 infection or if they did not describe the maternal-perinatal outcomes. Moreover, systematic reviews and those studies with patients from several countries were excluded. Additionally, we also excluded research works not showing a wide spectrum of COVID-19 and only focused on patients with severe morbidity to avoid a possible bias towards the severity of the disease. 

All articles were independently analyzed by two authors (YCL and OCV) and if the title and abstract did not provide useful information for the review or was irrelevant, the articles were eliminated from the analysis. Any disagreement in the eligibility of studies was resolved through discussion and joint assessment until consensus was reached between both researchers. 

The full text of the articles included in the systematic review was then obtained and evaluated and their references were also analyzed, looking for new articles to be included.

### 2.1. Data Collection and Data Items

Data collection was performed with a standard form. The variables that were collected for each article were: author; year of publication; starting and ending date of patient recruitment; design and type of study (single- or multi-center); city and country where the study was performed; total number of patients, number of patients with symptoms, and number of asymptomatic patients; universal screening [yes/no]; maternal characteristics including maternal age, use of tobacco, maternal morbidities, maternal body mass index (BMI), and nulliparous rate; obstetric characteristics including onset of symptoms in pregnancy or puerperium, gestational morbidities, gestational age (GA) at triage; complementary maternal studies as performing of the real-time reverse transcription-polymerase chain reaction (RT-PCR) and the results of the oral swab; type of radiological exam; pneumonia; analytical disorders as leukocytosis (defined as leukocytes count higher than 10,000 cells per cubic millimeter); lymphocytopenia (defined as lymphocyte count of less than 1500 cells per cubic millimeter); thrombocytopenia (platelet count of less than 150,000 per cubic millimeter); lactate dehydrogenase level (LDH) increased (higher than 250 U/L); maternal management including maternal treatment (antiviral, antibiotics, steroids, low molecular weight heparin and interferon), admission to intensive care unit (ICU), oxygen therapy, mechanical ventilation; and finally, maternal-perinatal outcomes as maternal mortality (MM), GA at delivery, mode of delivery (C-section or vaginal delivery), preterm birth (GA less than 37 weeks), admission to neonatal intensive care unit (NICU), neonatal birthweight, Apgar test value at 5 min, neonatal asphyxia, perinatal mortality, vertical transmission, and breastfeeding. 

A confirmed case of SARS-CoV-2 infection is defined as a positive result on high-throughput sequencing or RT-PCR assay of nasal or pharyngeal sway specimens. 

According to the seventh version of the guidelines on the Diagnosis and Treatment of COVID-19 by the National Health Commission of China [18], COVID-19 severity is classified as follows:

1. Mild cases: the clinical symptoms were mild and there was no sign of pneumonia on imaging.
2. Moderate cases: fever and respiratory symptoms with radiological findings of pneumonia.
3. Severe cases: any of the following conditions:
  - Respiratory distress [respiratory rate of ≥ 30 per min].
  - Oxygen saturation on room air at rest ≤ 93%.
  - Partial pressure of oxygen in arterial blood/fraction of inspired oxygen ≤300 mmHg.
4. Critical cases:
  - Respiratory failure and requiring mechanical ventilation.
  - Shock.
  - Patients with another organ failure that requires ICU care.

A descriptive study of all the articles included in the work was performed. Furthermore, we did an analytical study comparing the maternal and obstetric characteristics, the complementary maternal studies, the maternal management, and the maternal-perinatal outcomes based on the country of origin of the publication. Finally, graphical representations of the obtained results were carried out in each article regarding maternal perinatal variables such as proportion of C-section and prematurity, grouped according to the country of origin of the publication. 

### 2.2. Risk of Bias Assessment and Statistical Analysis

Risk of bias was assessed independently by both authors, determining the adequacy of compliance with the inclusion criteria. The quality of the evidence of studies included was assessed using the methodologic quality and synthesis of case series and case reports described by Murad MH et al [19]. Based on this tool, each study is judged on four perspectives: the selection of the study groups, the ascertainment, and the casuality of the outcome observed and the reporting of the case. The articles can reach eight stars: one in the selection and the reporting, two in the ascertainment, and four stars in the casuality [19,20]. 

The data obtained from the studies were included in a Microsoft Office Excel database, version 16.42 (Microsoft, Redmond, WA, USA) and the statistical analysis was performed with Stata 13.1 (StataCorp LLC, College Station, TX, USA). Differences with *p* < 0.05 were considered statistically significant. Quantitative variables were expressed as mean (interquartile range or 95% confidence interval (CI)) and categorical variables as the number of patients and rates (%) (CI95%). Univariate analysis was performed using Fisher’s exact test, chi-squared test, or Student’s *t*-test, as appropriate. 

## 3. Results

**Figure 1:**
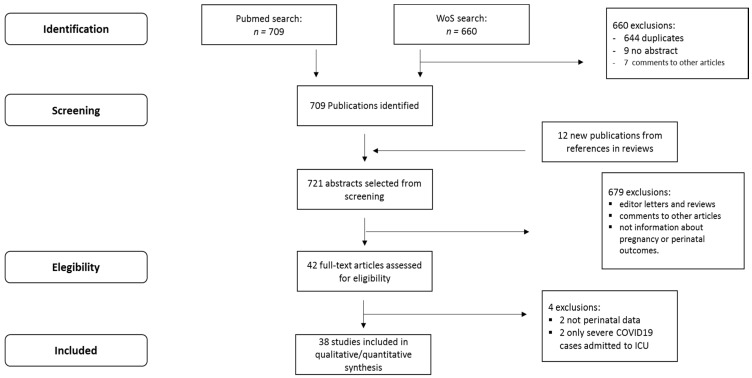
Flow chart of studies retrieved and included in the systematic review.

Figure 1 presents the flow chart of the SR. Of the articles identified in the 2 databases, a total of 38 articles [21,22,23,24,25,26,27,28,29,30,31,32,33,34,35,36,37,38,39,40,41,42,43,44,45,46,47,48,49,50,51,52,53,54,55,56,57,58] were included in this systematic review with 2670 patients proceeding from 7 countries (China, United States of America (USA), France, United Kingdom (UK), Spain, Italy, and Portugal).

The main variables from the studies are described in the Supplementary Table S1. Nineteen of the 20 works published by China (95%) have been carried out in Wuhan, within the province of Hubei. Six of the 7 studies from the USA (85.7%) come from the east coast of the country and 2 of the 4 studies from France (50%) come from Paris. All of them were published in 2020, with a maximum recruitment period of 3 months [21]. The first patient recruited in China was on 8 December 2019 [21] and the last on 24 March 2020 [22], while in the USA, the first patient was recruited on 21 January 2020 [45] and the last was on 24 April 2020 [43]. From the total articles, 34 (89.5%) were a case series and 20 (52.6%) were conducted in a single center. 

**Figure 2:**
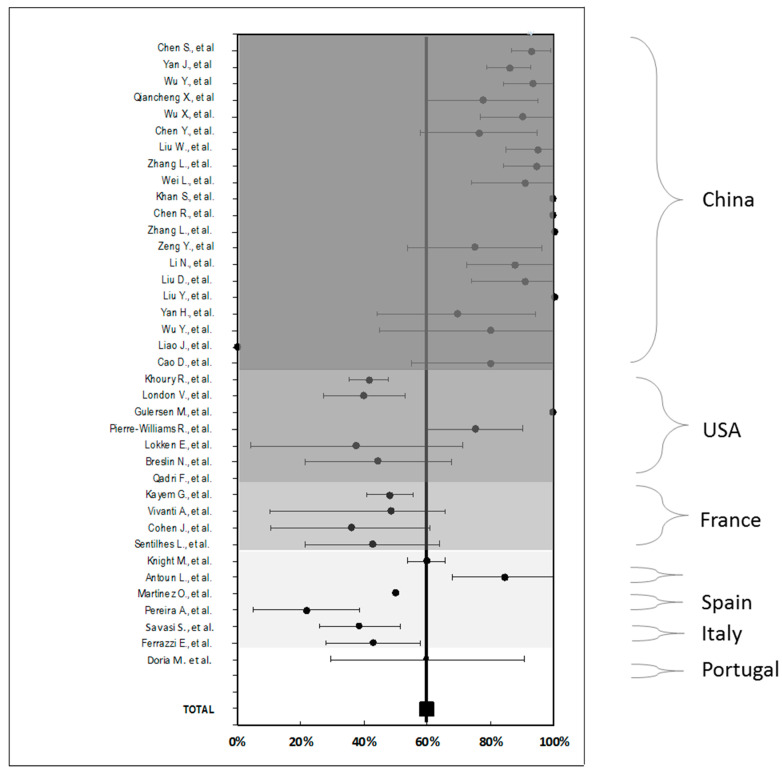
Percentage of C-section with 95% confidence interval observed in the studies and grouped by country.

Table 1 summarizes collected data of every article, distinguished by the country of origin of the publication according to maternal and obstetric characteristics, signs and symptoms reported, maternal outcomes, treatments, and perinatal outcomes. In this table, just as in Figure 2 countries, countries were represented from higher to lower number of articles identified by grayscales. 

China is the most representative country in our study with 20 works (52.6% of articles) and France is the region with more cases included; a total of 859 patients just on 2 articles (32.2% of the total cases) 

In the overall descriptive, taking into account the data obtained by the studies, we can report that the average patient was 31.4 years old and the median GA at the triage was 34.1 weeks. Up to 2170 patients (81.3%) presented subjective symptoms of COVID-19 in the triage, 71.3% clinical findings and/or radiological imaging related with pneumonia, and the median maternal ICU admission was about 6.1%. In all the pregnant women, proportion of C-section was of 67.1% and the maternal median mortality was of 2 per 1000. Regarding perinatal data, we reported 21.6% prematurity, with a perinatal mortality of 2 per 1000, being the probability of vertical transmission registered of 1.8%.

Afterwards, conducting a comparative analysis of the data described in Table 1, we observed significative statistic differences in respect of the number of symptomatic patients, percentage of smoker patients, maternal average age, proportion of patients with symptoms at triage, the type of radiological exam, quantity of patients with pneumonia, C-section delivery, maternal mortality rate, percentage of patients receiving treatments, antivirals, antibiotics or anticoagulant drugs, along with the median of new-born weight.

In Figure 2, it can be observed the proportion of C-sections reported in every work included in this systematic review and grouped according the country of origin. Weighted average as of all cases was calculated, with the percentage of C-section at 59.7%. As it is shown, China is the country with the most C-sections published (83.9%), followed by UK (71.9%), with Spain the region with the lowest rate of C-sections in their works (35.9%). 

**Figure 3:**
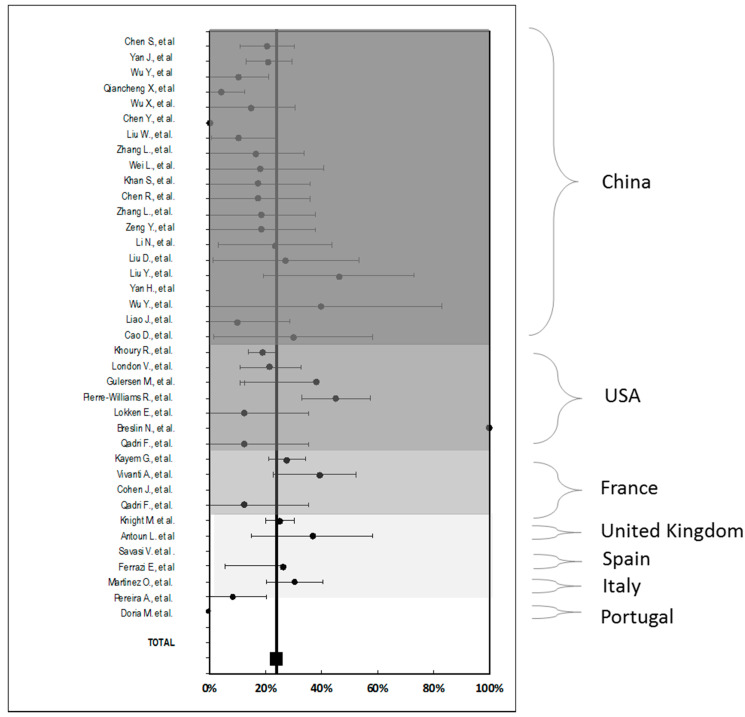
Percentage of prematurity, with 95% confidence interval observed in the studies and grouped by country.

Similar to the previous one, Figure 3 show the proportion of prematurity published in the selected articles in the systematic review, with its weighted mean at 23.9%. UK is the country with the highest number of preterm deliveries (31%), immediately followed by France (30.1%). It is of note that the series of cases proceeding from Portugal have not registered any case of prematurity. 

As a particular overview of the published works based on the country of origin and considering the results described in Supplementary Tables S1 and S2 as well as Figure 2 and Figure 3, it could be summarized that patients included in the works from China (52.6% of our study) were the first women recruited according to the evolution of this global pandemic, with the first case reported on 8 December 2019 [21]. Obstetric patients from China were among the youngest women (30.9 years) with a low BMI (22.8 kg/m2). Its patients are mostly nulliparous (51.7%), 1 in 3 with another obstetric morbidity (39.3%), and symptomatology in the triage up to 82% of the cases. In this region, just like France, elaborated radiological studies were done by using a chest CT scan in all cases (100%). Equally, in both countries the highest rate of patients with pneumonia was found (China 84.6% and France 83.3%), but they differed in the urgency of maternal ICU admission (China 1.8%; France 8.7%) and the necessity of respiratory support (China 1.8% and France 6.4%). Series from China have reported on average the highest percentage of antiviral treatments (75.4%) and antibiotics (84%), together with the highest proportion of C-sections (83.9%). What is more, despite reporting the lowest rate of prematurity (19.3%), NICU admission is the largest of the different countries included in this systematic review (54.6%). Comparable to the rest of the regions, newborns obtained an optimum perinatal result with low rates of perinatal mortality (0.1%) and vertical transmission (2.0%).

Works published from the USA described a patient with the highest BMI levels (31.8 kg/m2) as well as maternal comorbidities (32.6%). In this country, median GA at triage (31.5 weeks) and the average percentage of patients with symptoms at the triage (60.8%) was the lowest of the registered in this systematic review. After China, it reported the highest rate of patients with urgency of maternal oxygen therapy (38.4%) and NICU admission to newborns (35.3%). 

As we previously reported, despite only reporting 4 articles, France contributed to the most number of cases in this systematic review (859 cases, 32.2% of the total series). In this country, the lowest rate of maternal comorbidities was described (14.8%), followed by obstetrics (10.6%) and nulliparity (27.3%). In addition, 85.9% of the patients were symptomatic and this is the third country with the highest proportion of pneumonia (83.3%) after UK (100%) and China (84.6%). Looking at the maternal treatment, it is of note that the percentage of the antivirals (5.6%) and antibiotic (7.4%) were one of the lowest and no patients required corticotherapy. France is the second country regarding prematurity (30.1%) closet o UK (31%), reporting lower birthweight (2800 gr) followed by the USA (2769 gr).

UK, similar to Italy and Spain, only provides 2 works (5.6%), within a total of 450 patients (16.9% of the total series), collecting the last patient recruited on 30 April of 2020 [43]. Although the median maternal age in this country is the lowest (29.8 years), it is the second country following Italy (14.6%) with the highest maternal admission in the ICU, and after China (83.9%) in the proportion of C-sections (71.9%). We would like to highlight that this country is the first that looked at the need of respiratory support (11.5%) and maternal mortality (2.8%). 

As abovementioned, either the Spain or Italy count with 2 works, we observed the eldest patients in these countries (34.6 vs. 33.0 years, respectively), and around 1 in 3 maternal comorbidities (39.2% vs. 32%). The percentage of patients with symptoms is really close (73.9% vs. 83.2%), and however, in Italy, the percentage of patients with pneumonia and transfer to ICU is virtually twice the total of Spain (29% vs. 57.6% and 6.4% vs. 14.6%, respectively). Both countries reported the lowest rates of C-sections in our systematic review (35.9% in Spain vs. 40.8% in Italy), although in Spain, the probability of NICU admission was higher (26.8%) than Italy (8.1%) even though similar results were obtained in the percentage of prematurity (19.6% in Spain vs. 23.6% in Italy). The rate of vertical transmission found in Italy was the highest in our study (3.6%) and like Spain and Portugal, there were no perinatal mortality cases reported. 

Portugal only afforded an article with 12 patients (0.4% of the total patients), with little data available collected in this systematic review. Nonetheless, it is the country with the highest rate of obstetric morbidity (41.7%) and the largest GA at triage (37.5 weeks).

Finally, the results about the quality of the evidence of studies could be visualized in the Supplementary Table S2. Thirty-five of the works (92.1%), were catalogued with 5 stars and 3 of them (7.9%) from China, USA, and UKA with 6 stars out of 8, the maximum value reached following the methodologic quality and synthesis of case series and case reports described by Murad MH. et al. [19]. 

## 4. Discussion

This systematic review has finally included 38 works and 2670 pregnant women infected by SARS-CoV-2, collected from 7 different countries. Because of the great scientific interest provoked by the pandemic during the last months, multiple systematic reviews have been published with a noteworthy casuistic, such as those conducted by Khalil et al. [59] with 2567 pregnancies and Diriba et al. [60] with 1360 patients. Notwithstanding, our work provides the largest number of obstetric patients issued until the date, along with disclosing the profile of the patients according to the country of origin. 

Subsequently, to the analysis of the results, we can establish that published cases partly agree with the worldwide suffering caused by this pandemic, being China the first country regarding the recruitment and publications available until the date. In addition, the publications included in this review come mainly from the cities or geographic areas most affected by the pandemic within each of the countries, such as is the case of Wuhan, China, the east coast of the USA, or Madrid in Spain. Furthermore, data collected in this systematic review guide us in noticing that patients have been recruited during the first wave in these 7 countries, firstly because of the short recruitment period (maximum 3 months in the work conducted by Chen et al. [21] and the final date of the collection period, at the end of April in the work conducted by Antoun et al. [43]. 

With the exception of China, which has had a recruitment period of almost 4 months, the rest of the countries in this review do not exceed two months. In addition, the USA began its recruitment on 21 January and France on 1 March 2020, which has been 40 days and 80 days after the start in China, respectively. This could also explain differences between countries, for instance, in the wide spectrum of treatments used or in the C-section rates, such as, for example, in the percentage of C-sections since studies with earlier recruitment, between December 2019 and February 2020, the proportion of C-sections is higher (between 77.3% [24] and 100% [28,30,31]). compared to those countries with a later recruitment, between March and April 2020 (between 33.3 % [47] and 75% [44]).

Henceforth, it is possible that because of the rapid expansion of the pandemic, the next works pending publication may be located in the first wave of the pandemic in those countries still with no data reported or maybe they could be overlapped with patients during the second wave of the pandemic. The interest in the publications and in the knowledge of the various affectations in pregnant women affected by SARS-CoV-2 infection to respond to the different professionals how to manage the infection in the maternal perinatal duality, may be concurrent with the proper geographic expansion globally. We hope that more countries could share their results to enrich this important issue. 

As previously indicated, the countries with the highest number of articles contributed to this review is China (20 works, 52.6% of the total) and the country with the largest number of patients is France (859, 32.2% of the patients). It is a fact that, until 30 April, China reported 84,773 confirmed cases with 4643 deaths, whereas France registered 127,066 cases and 24,054 deaths by COVID-19 [61]. 

Despite knowing how SARS-CoV-2 has spread worldwide from the beginning of the pandemic, this systematic review does not include pregnant women from other countries hard hit by this situation like Brazil, Colombia, Mexico, India, Russian Federation, Argentina, or Iran. A possible explanation of this fact is that the series of cases in these countries have been published collectively in various multinational studies as it is the case of the WAPM study, with a total of 388 pregnant women from 73 different centers from 22 different countries [62,63]. Another likely reason is that only the most severe cases have been reported, with the worst maternal perinatal outcomes, leading to a selective report bias, as it is the case of the 20 COVID-19-related maternal deaths found by Takemoto et al. [64] in Brazil, the 10 maternal deaths in Mexico [65], and the 9 cases of maternal defunction in Iran [66]. 

Global results obtained in this systematic review highlight a mild increase in the maternal age rate in the majority of the studies (31.4 years), with remarkable data originating from Spain, France, and even the USA if we consider the median age recorded by the Statistic Office of the European Union (Eurostat) 2017 which was 30.6, 32.1, or 29.1 years, respectively [67]. This augmentation in the maternal age of our series could be instead associated with the increased maternal morbidity observed, according to the studies of Aoyama et al. [68] and Lisonkoya et al. [69]. 

Regarding maternal clinical, up to 81.3% of patients reported symptoms whose distribution contrasts with the published data in series of non-expectant adults [6] and this is fundamentally due to the case of publication bias and the own recruitment period of the first wave, with almost no universal screening of SARS-CoV-2 in obstetric patients. We intend to highlight this fact, as it may aid explaining part of the differences detected in the profile of patients according to the country of origin. The high proportion of symptomatic patients may conduct worse maternal perinatal results, with a higher rate of preterm delivery and need for respiratory support than asymptomatic pregnant women, as stated by London et al. [45]. 

**Figure 4:**
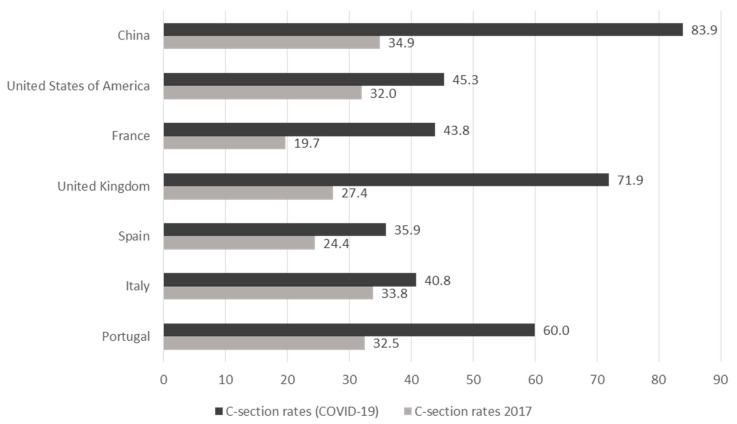
C-section rates in 2017 by countries [70] compared with C-section rates registered in the systematic review.

The proportion of C-sections (67.1%) observed in overall cases and individually for countries was superior to the previously reported. The comparison between the proportion of C-sections in 2017 published by country [70] versus that obtained in this systematic review can be seen in Figure 4. 

Differences in the proportion of C-sections between countries may be due to variability in pre-pandemic obstetric management. The increase observed during the pandemic is proportional to these differences, highlighting the case of China, with an increase of 49%, higher than expected, probably due to its earlier recruitment period, ignorance about the pathophysiology of COVID-19, and the possible risk of vertical transmission. These facts could be corroborated in the incidence of prematurity, so future studies should study this same trend in the probability of preterm births. 

Similarly, described overall [21.6%] and individual prematurity were strongly superior to the work published by Chawanpaiboon S. et al. reporting a 10.6% worldwide, oscillating among 7.0% in Spain at 2014, 6.3% in the UK, 9.9% in the USA, or 13.4% in Northern Africa [71]. The increase in the maternal and perinatal morbidity could be related to the quick respiratory worsening of the mother, which forces an urgent and/or a planned delivery during the first wave of the pandemic [72], which was significative. The acquired knowledge about the pathophysiology and management of SARS-CoV-2 infection and the higher number of diagnoses in asymptomatic patients will probably result in a decrease in the percentage of C-sections and prematurity in future works. 

As we have previously discussed, Spain presented the population of patients with the highest maternal age (34.6 years). Spain has one of the lowest birth rates in the world and this can be attributed to the economic crisis that occurred in the 1970s, as well as to changes in the behavior and habits of Spanish couples that persisted despite improvements in the country´s economy in later decades [73,74]. USA is the country with the highest BMI compared with other countries like China or Italy. These data agree with the published global information, as in 2016, the mean BMI in women in USA was 29.1 kg/m2, different than 23.6 kg/m2 and 24.9 kg/m2 of China and Italy, respectively [75]. This is an important fact as the augmentation in the BMI is closely related with worse prognosis and for non-expectant population, patients with BMI >25 kg/m2 have a probability 4 times superior to perish by COVID-19 [76]. With the obtained data of our work, we cannot establish a relation of causality between obesity and worsening maternal perinatal outcomes, although available evidence suggests that SARS-CoV-2 infection may not behave as mild as suggested during pregnancy, especially with factors such as obesity [77].

It is of note that all Chinese and French patients were evaluated by CT scan as radiological tests. Borakati et al. [78] studied the diagnostic accuracy of X-ray versus CT in COVID-19. Following these authors, the sensitivity and specificity of CXR for COVID-19 diagnosis were 0.56 and 0.60 and for CT scans, 0.85 and 0.50, respectively, concluding that CT has substantially improved diagnostic performance over CXR and CT should be strongly considered in the initial assessment for suspected COVID-19 [78]. This could be due either for the higher availability to perform chest CT in the various countries previously commented, along with the novelty suffered by China, as they were the first to face a new and unknown disease, therefore precise radiological tests with higher diagnostic accuracy. 

Another distinctive variable is the proportion of patients with pneumonia as UK, China, and France reported higher cases when compared to Italy, USA, and Spain. Although we ignored the real motifs of these findings, it could be associated with the differences in the definition on criteria for pneumonia in patients with COVID-19 and even with the diagnostic accuracy of the imaging technique undergone, as those countries where CT scans were conducted also showed the highest risk for suffering pneumonia. 

When analyzing maternal outcomes, we observed relevant clinical differences although some events of severe morbidity, such as urgency of respiratory support and maternal admission in the ICU, as the UK showed notably increased values in comparison to China (11.5% and 13.7% vs. 1.8% and 1.8%, *p* = 0.393, and *p* = 0.093); maternal mortality is also superior in UK against the global series (2.8% vs. 0.2%, *p* = 0.000). These findings should be taken cautiously, as it may promote a publication bias exclusively with negative results of severe and critical cases reported [79,80].

Likewise, we have found differences regarding the administration and type of maternal treatments. China is the country with the most therapies used (95.6% of their patients), mainly antivirals and antibiotics, contrary to the USA, only administering drugs to 1 out of 10 patients, mainly anticoagulants. This may be due to the unknowledge of either therapeutic chances (as COVID-19 is a novel disease which many changes reported in the pharmacological management) or the temporary period in which the pandemic has affected the different countries. 

Following the maternal perinatal results as observed in Figure 2, the proportion of C-sections was different among countries, as China and UK reported higher values compared to those obtained globally, whereas France, Italy, and Spain published lower proportions to the total of the series. As discussed before, this could be due to the habitual obstetric practice of each region, as the differences in the severity of COVID-19 reported in each work, as it seems that these high C-section rates do not seem to be representative of women who have mild to moderate disease [81].

Finally, as represented in Figure 3, relevant clinical differences were observed without reaching statistical significance in the proportion of prematurity as UK and France and UK present higher rates than Spain or Portugal. It is possible that many of the preterm deliveries were iatrogenic, for maternal reasons or fetal distress. At present, there is insufficient evidence to determine any correlation between spontaneous preterm labor and COVID-19 infection in pregnancy although there are some reported cases of preterm prelabor rupture of membrane [81,82].

Main strengthens of this systematic review is that it comprises the highest revision of pregnant women infected by SARS-CoV-2 until the date and it is the first work in studying and comparing the profile of obstetric patients according to the country of origin. Furthermore, as underlined, despite the impossibility of collecting studies original from other countries, the results seem to be concordant with the expansion during the first wave of the seven countries and this information can help to know how the countries have approached maternal and perinatal health during the pandemic.

Regarding the limitations of our study, it is of note that most conclusions extracted are based on published data, without precisely knowing the real percentage of patients not belonging to the country of publication, being influenced by some other variable described. 

As we have indicated in the results, and despite the fact that the main objective of the review was to explain the specific profile of each of the countries of origin of the publication, the works come from very specific areas within each territory, so possibly, do not represent the global dimension of the country.

Moreover, when analyzing the proportion of C-sections, we ignore the percentage of patients with previous C-sections or other conditions that may increase the risk of going through this procedure. In addition, because of the essence of the primary studies, it is likely that publication bias may appear, as we only included works published from two databases, selection, and report result bias. 

Within these biases and because the exclusion criterion of studies with only severe cases of COVID-19 has been used, there may be a loss of the severity dimension of this pathology. Nor has this study had the objective of collecting and independently analyzing the effect of other maternal variables such as the maternal socioeconomic status (for example, education level or household income) or healthcare access, data that could explain the possible differences observed in the patterns from each of the countries. These variables would likely influence general maternal health and disease risk, giving rise to a research path for future work. 

Despite current knowledge that the affectation by SARS-CoV-2 is uniform worldwide, it is unknown if differences inside the own virus may explain the distinct results found in the various geographic regions. 

Moreover, it is possible that we could not include some works currently writing or published after the death line selected in this review. 

## 5. Conclusions

It is of note that pregnant women infected by SARS-CoV-2 reported during the first wave of the pandemic, either globally or the majority of countries included in this systematic review, are patients with higher median age compared in her own country, with increased percentage of obesity, baseline and pregnancy comorbidities, mainly symptomatic during the third trimester of pregnancy, requiring hospitalization and maternal therapy, although with a low proportion of ICU admission and very rare maternal mortality. When analyzing delivery, patients present a high rate of C-sections and prematurity, probably related to the rapid worsening of the maternal status, as newborn results described in general were favorable.

Differences found in the maternal characteristics according to the country of origin could be sustained on the proper characteristics of the pregnant women in each country (maternal age, BMI, or percentage of comorbidities), the strategy of applying universal or clinical screening at the moment of patient recruitment as well as the initial management of the therapeutic arsenal, always in agreement with the current knowledge of the disease.

The findings found in this review are relevant to public health and healthcare professionals. Among other causes, we have described the possibility that the pre-pandemic patterns are part of the cause of the differences observed during the pandemic, but also the baseline situation of the patients in each region, the period of time that the pandemic affects each region of the territories, and the advance in the knowledge of the management tools that have emerged during our study period. Therefore, new lines of research are suggested that, together with the information obtained in this work, can help us to know how different countries have approached the maternal and perinatal health during the COVID-19 pandemic and identify effective healthcare strategies.

## Figures and Tables

**Table 1:** Description of the main variables in the overall series and comparison of the results by countries.

	Number (%) of Patients Reporting Results	Overall	China	United States of America	France	United Kingdom	Spain	Italy	Portugal	*p* Value
*n*, %	2670 (100)	2670	545 (20.4)	543 (20.3)	859 (32.2)	450 (16.9)	142 (5.3)	119 (4.5)	12 (0.4)	
Articles, *n*, %	38 (100)	38	20 (52.6)	7 (18.4)	4 (10.5)	2 (5.6)	2 (5.6)	2 (5.6)	1 (2.6)	
Patients with symptoms, *n*, %	2170 (100)	2170 (81.3)	447 (82.0)	330 (60.8)	738 (85.9)	450 (100)	105 (73.9)	99 (83.2)	1 (8.3)	0.0112
Asymptomatic patients, *n*, %	492 (100)	492 (18.4)	90 (16.5)	213 (39.2)	121 (14.1)	0 (0.0)	37 (26.1)	20 (16.8)	11 (91.7)	NS
**Maternal characteristics**										
Maternal age, mean, CI 95%	1610 (60.3)	31.4 (30.9–31.9)	30.9 (30.3–31.4)	31.4 (30.1–32.8)	32.3 (30.3–34.2)	29.8	34.6 (34.3–34.7)	33.0 (32.0–33.9)	32.4	0.005
Tobacco, median, IQR	1064 (39.9)	2.9 (1.0–4.8)	n.r.	2.2	2.5 (1.6–3.3)	n.r.	7.3	1	n.r.	0.022
BMI, mean, CI 95%	713 (26.7)	26.2 (23.6–28.8)	22.8 (20.6–25.0)	31.8 (30.5–33.1)	25 (22.3–27.7)	n.r.	24.4	22.8	n.r.	NS
Maternal morbidities, mean, CI 95%	2143 (80.2)	25.7 (18.3–33.0)	20.9 (5.9–35.9)	32.6 (21.0–44.2)	14.8 (5.9–23.8)	34.0	39	32.0	41.7	NS
**Obstetric characteristics**										
Nulliparous, mean, CI 95%	1528 (57.2)	39.7 (32.0–47.4)	51.7 (34.5–68.7)	29.7 (26.4–33.0)	27.3	37.5	38.4 (24.3–52.4)	37.9 (33.4–42.4)	n.r.	NS
Obstetric morbidities, mean, CI 95%	2194 (82.2%)	30.6 (20.7–40.5)	39.3 (25.6–53.1)	13.2 (7.4–19.0)	10.6 (5.1–16.0)	30	12.2	14	83.3	NS
Gestational age at triage, mean, CI 95%	1334 (50.0)	34.1 (32.2–36.1)	35.9 (32.8–39.0)	31.5 (27.1–35.9)	30.2 (26.5–34.0=	33.5 (32.4–34.5)	32	37	37.5	NS
**Complementary maternal studies**										
Symptoms at triage, mean, CI 95%	2654 (99.4)	78.6 (70.8–86.4)	79.8 (70.9–88.7)	67.2 (46.3–88.2)	94.9	100	74.1 (72.3–75.9)	92	8.3	0.008
Radiological exam	2645 (99.1)									<0.001
CT, *n*, %		1391 (52.6)	532 (100.0)	0 (0.0)	859 (100.0)	0 (0.0)	0 (0.0)	0 (0.0)	n.r.	<0.001
Chest X-r		1254 (47.4)	0 (0.0)	543 (100.0)	0 (0.0)	450 (100.0)	142 (100.0)	119 (100.0)	n.r.	<0.001
Pneumonia, mean, CI 95%	1305 (48.9)	71.3 (58.5–84.1)	84.6 (71.9–97.4)	34.7 (0.9–68.4)	83.3	100	29 (27.0–31.0)	57.6 (32.2–83.0)	n.r.	0.011
Leukocytosis, mean, CI 95%	407 (15.2)	31.4 (18.1–44.7)	32.7 (17.7–47.8)	n.r.	n.r.	n.r.	10	38.2	n.r.	NS
Lymphopenia, mean, CI 95%	993 (37.2)	35.9 (26.8–44.9)	37.5 (24.3–50.7)	40.1 (25.4–54.7)	40.3 (31.8–48.8)	n.r.	24.3	26.6 (21.6–31.6)	n.r.	NS
Trhombocitopenia, mean, CI 95%	267 (10)	8.2 (−4.0–20.3)	10.5 (−9.9–30.9)	n.r.	n.r.	n.r.	15	n.r.	n.r.	NS
Elevated LDH, mean, CI 95%	273 (10.2)	28.2 (16.4–40.0)	27.9 (13.2–42.5)	n.r.	n.r.	n.r.	20	39	n.r.	NS
**Maternal management**										
Maternal treatments, mean, CI 95%	380 (14.3)	86.0 (69.4–102.6)	95.6 (90.0–101.3)	11.6	n.r.	n.r.	65	n.r.	n.r.	<0.001
Antiviral, mean, CI 95%	1040 (39.0)	52.1 (33.4–70.6)	75.4 (57.8–93.0)	14.7 (3.2–26.2)	5.6	2	18.3	38	n.r.	0.011
Antibiotic, mean, CI 95%	752 (28.2)	55.4 (36.6–74.1)	84.0 (66.0–102.0)	20.8 (4.6–37.0)	7.4	n.r.	65	43	n.r.	0.002
Steroids, mean, CI 95%	919 (34.4)	17.2 (10.4–24.0)	21.0 (9.1–32.8)	13.9 (3.2–24.6)	0	20.7 (8.3–33.0)	15	n.r.	n.r.	NS
Anticoagulants, mean, CI 95%	222 (8.3)	39.9 (−5.1–84.9)	4.8	74	n.r.	n.r.	41.7	39	n.r.	<0.001
Admission to ICU, mean, CI 95 %	1664 (62.3)	6.1 (2.9–9.3)	1.8 (−0.1–3.7)	9.1 (−2.7–20.8)	8.7 (7.3–10.0)	13.7 (6.1–21.3)	6.4 (−3.2–15.9)	14.6 (1.0–28.1)	n.r.	NS
Oxygen therapy, mean, CI 95 %	1368 (51.2)	37.8 (20.4–55.2)	63.6 (29.7–97.5)	38.4 (−7.0–83.9)	14.1 (6.1–22.0)	n.r.	11.7 (8.1–15.3)	32.9 (24.6–41.2)	n.r.	NS
Mechanical ventilation, mean, CI 95 %	2254 (84.4)	4.6 (1.8–7.4)	1.8 (−0.1–3.7)	7.5 (−4.1–19.2)	6.4 (3.7–9.1)	11.5 (8.4–14.6)	3.7 (−3.9–11.2)	3.9	n.r.	NS
**Maternal-Perinatal outcomes**										
Maternal mortality, mean, CI 95%	2523 (94.5)	0.2 (−0.1–0.4)	0.0	0.0	0.1 (−0.1–0.2)	2.8 (−0.4–5.9)	0.0	0.0	0.0	0.000
GA at delivery, mean, CI 95 %	1441 (54.0)	37.9 (37.4–38.3)	37.9 (37.5–38.3)	37.3 (34.4–40.2)	37.7 (37.1–38.2)	38	37.6	39	n.r.	NS
C-section, mean, CI 95 (%)	2605 (97.6)	67.1 (58.3–75.9)	83.9 (74.0–93.8)	45.3 (33.8–57.7)	43.8 (37.7–49.9)	71.9 (46.8–96.9)	35.9 (7.2–64.5)	40.8 (36.4–45.1)	60	0.000
Prematurity (< 37 w), mean, CI 95 %	2526 (94.6)	21.6 (17.6–25.6)	19.3 (14.2–24.5)	24.9 (13.3–36.4)	30.1 (20.9–39.4)	31 (19.2–42.8)	19.6 (−2.6–41.8)	23.6 (18.3–28.9)	0.0	NS
Admission to NICU (%)	1982 (74.2)	36.4 (19.7–53.0)	54.6 (20.9–88.2)	35.3 (4.9–65.8)	20.6 (12.1–29.1)	25.3	26.8	8.1 (6.0–10.1)	n.r.	NS
Birthweight, mean, CI 95%	921 (34.5)	3063.5 (2974.5–3152.4)	3138.2 (3089.0–3187.4)	2769 (2007.9–3530.1)	2800	3139	3049	3122 (3043.0–3201.0)	2691	0.033
Apgar 5 minutes, mean, CI 95%	886 (33.2)	9.4 (9.1–9.7)	9.5 (9.2–9.8)	8.7 (8.1–9.3)	n.r.	9.3	n.r.	10	9.9	NS
Perinatal mortality, mean, CI 95%	2501 (93.7)	0.2 (0.0–0.3)	0.1 (−0.1–0.2)	0.5 (−0.2–1.2)	0.4 (−0.4–1.3)	0.5 (−0.5–1.5)	0	0	0	NS
Vertical transmission, mean, CI 95%	2471 (92.5)	1.8 (0.0–3.6)	2.0 (−1.0–5.0)	1.1 (−0.3–2.5)	1.4 (−0.4–3.1)	2.5 (−2.6–7.6)	1.2 (−1.2–3.6)	3.6 (−3.7–10.8)	0.0	NS
Maternal breastfeeding, mean, CI 95%	290 (10.9)	63.1 (34.0–92.2)	60	50	n.r.	n.r.	71.3 (19.7–122.8)	63.1	n.r.	NS

## Data Availability

The data presented in this study are available on request from the corresponding author.

## Supplementary Materials

The following are available online at https://www.mdpi.com/2077-0383/10/2/360/s1, Table S1. Main variables from the studies, Table S2: Reporting studies according to countries.
